# Topical emollient therapy in the management of severe acute malnutrition in children under two: A randomized controlled clinical trial in Bangladesh

**DOI:** 10.7189/jogh.10.010414

**Published:** 2020-06

**Authors:** KM Shahunja, Tahmeed Ahmed, Md Iqbal Hossain, Mustafa Mahfuz, Lindsay Kendall, Xinyi Zhu, Krishan Singh, Jonathan M Crowther, Sunita Singh, Rachel A Gibson, Gary L Darmstadt

**Affiliations:** 1 Nutrition and Clinical Services Division, International Centre for Diarrhoeal Disease Research, Bangladesh (icddr,b), Dhaka, Bangladesh; 2 GlaxoSmithKline R&D, Gunnels Wood Road, Stevenage, Hertfordshire, UK; 3 JMC Scientific Consulting Ltd, Egham, Surrey, UK; 4 Department of Pediatrics, Stanford University School of Medicine, Stanford, California, USA

## Abstract

**Background:**

Topical emollient therapy can improve neonatal health and growth and potentially provides an additional avenue for augmenting the provision of nutrition to children with severe acute malnutrition (SAM). We hypothesised that topical treatment of hospitalised children with SAM using sunflower seed oil (SSO), in addition to standard-of-care for SAM, would improve skin barrier function and weight gain, reduce risk of infection, and accelerate clinical recovery.

**Methods:**

We conducted a randomised, two-arm, controlled, unblinded clinical trial in 212 subjects aged 2 to 24 months who were admitted for care of SAM at the ‘Dhaka Hospital’ of icddr,b during January 2016 to November 2017. Enrollment was age-stratified into 2 to <6 months and 6 to 24 months age groups in a 1:2 ratio. All children received SAM standard-of-care, and the SSO group was also treated with 3 g of SSO per kg body weight three times daily for 10 days. Primary outcome was rate of weight gain over the 10-day study period. Secondary endpoints included rate of nosocomial infection, time to recovery from acute illness, skin condition score, rate of transepidermal water loss (TEWL) and C-reactive protein (CRP) level.

**Results:**

Rate of weight gain was higher in the SSO than the control group (adjusted mean difference, AMD = 0.90 g/kg/d, 95% confidence interval (CI) = -1.22 to 3.03 in the younger age stratum), but did not reach statistical significance. Nosocomial infection rate was significantly lower in the SSO group in the older age stratum (adjusted odds ratio (OR) = 0.41, 95% CI = 0.19 to 0.85; *P* = 0.017), but was comparable in the younger age stratum and overall. Skin condition score improved (AMD = -14.88, 95% CI = -24.12 to -5.65, *P* = 0.002) and TEWL was reduced overall (AMD = -2.59, 95% CI = -3.86 to -1.31, *P* < 0.001) in the SSO group. Reduction in CRP level was significantly greater in the SSO group (median: -0.28) than the control group (median 0.00) (*P* = 0.019) in the younger age stratum.

**Conclusions:**

Topical therapy with SSO was beneficial for children with SAM when applied as adjunctive therapy. A community-based trial with a longer intervention period is recommended to validate these results.

**Trial registration:**

ClinicalTrials.gov: NCT02616289

Extreme poverty has declined significantly over the last two decades [1], yet child malnutrition remains a major public health concern in Asia and Africa. Severe acute malnutrition (SAM), defined as weight-for-length (or height) z-score <-3, is a major cause of mortality and morbidity among children under the age of 5 globally, particularly in South Asia and sub-Saharan Africa[2]. In 2014, an estimated 16 million children had SAM worldwide [2], and SAM caused about half a million under-five deaths [3]. In Bangladesh, approximately 600 000 children under five have SAM and close to 2 million have moderate acute malnutrition (-2 to -3 standard deviation of the median weight for length/height) [4]. When coupled with infectious illnesses, SAM increases the risk of under-five child death 9-fold [5].

The presentation of SAM in children includes compromised skin barrier function – sometimes manifest with hypo- or hyperpigmented scaling, exudative plaques and/or ulcerations – which increases risk for infection through portals of entry in the skin [6]. Essential fatty acids (EFAs) are important for skin barrier integrity but research from rural Bangladesh showed very low levels of EFAs in young children in the general population [7]. It is believed that EFA levels are further depleted in children with SAM.

Emollients are effective moisturisers that can help maintain the skin’s natural protective barrier and prevent water and heat loss through the skin. Research has demonstrated that applications of EFA-containing emollients (eg, sunflower-seed oil, SSO) augment skin barrier function, reduce transepidermal water loss (TEWL), protect from bloodstream infection and mortality, and promote weight gain in preterm neonates [8-12]. Oil massage in newborn infants in South Asia has also been reported to improve thermoregulation [13], increase serum triglyceride levels [14], and potentially improve neurodevelopment [15]. The cost of topical emollient therapy for management of hospitalised preterm infants in Bangladesh was an estimated US$ 61 per death averted and US$ 2.15 per year of potential life lost (YLL) averted (9.77 international dollars, I$, per YLL averted) [16].

To improve weight gain in children with SAM, management guidelines have focused on rehabilitation of children through oral and /or gastric feeding. However, there are diminishing returns on attempts to further improve protocols for feeding, and efforts are focused on incorporation of local foodstuffs into feeding protocols [17-19]. Emollient therapy provides an additional avenue for augmenting the provision of nutrition to children with SAM. Emollients such as SSO were utilised before the advent of total parenteral nutrition to provide nutrition via the skin to patients to prevent and treat EFA deficiency [20]. Acceleration of weight gain and increased weight at the end of the neonatal period have been documented in preterm infants on emollient therapy, and emollient therapy also leads to weight gain in children with disorders of the skin barrier such as severe eczema [12]. Emollient therapy not only provides nutrition through absorption of fatty acids but can also provide metabolic building blocks and up-regulate systems for skin barrier development, and conserve energy through reduced TEWL, reduced demands for maintenance of skin integrity and repair of damaged skin, and reduced immune system activation through reductions in infections due to improved skin and possibly gut barrier function [21-23].

We hypothesised that topical emollient therapy would improve skin barrier function and that essential and non-essential fatty acids would be absorbed through the skin, leading to reduced incidence of infections and acceleration of weight gain and clinical rehabilitation of young children with SAM.

## METHODS

### Trial registration

The trial was registered with ClinicalTrials.gov. Identification number: NCT02616289.

### Study design and participants

This study was a randomised two-arm, controlled, proof-of-concept clinical trial in Bangladesh. Participants were recruited at ‘Dhaka Hospital’ of the International Centre for Diarrhoeal Disease Research, Bangladesh (icddr,b), the largest diarrheal disease hospital worldwide, where more than 140 000 patients are treated annually free-of-cost. The majority of patients are under five years old and from low-income families. The hospital has evidence-based management guidelines [24] for children with SAM and a state-of-the-art inpatient nutrition rehabilitation unit which annually treats about 1500 under-five children.

Boys and girls aged 2 to 24 months were prospectively enrolled after admission to the icddr,b hospital with a diagnosis of SAM [weight-for-length z-score <-3 based on World Health Organization (WHO) growth standards [17], with complications such as diarrhea, pneumonia, etc, with or without nutritional edema] during January 2016 to December 2017. Participation was voluntary, and for all enrolled children written informed consent was obtained from the caregiver/legal guardian prior to participation. Other inclusion criteria included ability to comply with an inpatient stay of at least 10 days – the course of usual hospital treatment for SAM – and to suspend usual home skin care treatments during this time. Exclusion criteria included: the child was in custodial care (no longer looked after by their parent or legal guardian); the child presented with any life-threatening health condition such as septic shock and altered consciousness on admission; congenital disorders (eg, congenital heart disease, known metabolic disorders, chromosomal abnormalities, etc.); any known chronic disease (eg, tuberculosis), or any known history of allergy to emollient therapy.

### Randomisation and masking

Following admission to the icddr,b, participants meeting the inclusion criteria and providing informed consent were enrolled into the study. Participants were randomised in a 1:1 ratio (permuted block size of 4) to receive either topical emollient therapy together with standard-of-care or standard-of-care only for the 10-day duration of the study period. Permuted blocks for randomisation were prepared by a statistician who had no other role on this project. Individual randomisation codes were placed in sealed and opaque envelopes, concealed from the study team who had no role in the randomisation process. Only assigned study physicians or research assistants involved in recruitment had knowledge of the randomisation process and allocation of treatment arms. After enrolling a patient, the staff tore off the sequentially numbered (according to the serial of enrollment) sealed envelope and assigned the intervention arm according to the code inside. Randomisation of participants was stratified by age (2 to <6 months and 6 to 24 months) in a 1:2 ratio, respectively. The trial was an open-labeled study; due to the nature of the topical emollient intervention, masking was not possible.

### Treatment procedures

Following enrollment and initiation of hospital standard-of-care management, socio-demographic information, a detailed clinical history, a clinical examination and biological samples were obtained. Standard-of-care treatment was divided into two stages. Initial ‘acute phase’ treatment aimed to rescue the child from complications of their acute illnesses, followed by ‘rehabilitation phase’ management in order to achieve catch-up growth by giving emphasis on diet. Details about the SAM management protocol have been described elsewhere [24].

In addition to standard-of-care, participants randomised to the emollient therapy group were treated topically with emollient (3 g/kg body weight per dose three times daily at 9 am, 5 pm and 11 pm) for 10 days. Emollient therapy consisted of cold-pressed linoleic acid-rich (48% to 74%), low oleic acid (14%-39%) containing SSO donated by a commercial supplier (Cargill Refined Oils, Europe) (see Table S1 in the **Online Supplementary Document** for a full specification of the SSO). The oil was stored at -20°C in aliquots and defrosted to reach room temperature before use. The oil was then maintained at room temperature for up to 7 days after which it was replaced with fresh oil. Emollient was applied by dedicated study nurses trained in an appropriate method of gentle massage to ensure consistent application across all participants without injury to the skin. Oil was not applied to the face (to avoid the possibility of any accidental aspiration or ingestion), ears or scalp. The child’s anal area (to which the oil was applied last) was washed prior to application to avoid the spread of fecal flora. Bathing was delayed for a minimum of 3 to 4 hours after application of emollient to allow for absorption of oil. Any boils/pustules or infected skin were avoided during the massage process. The control group did not receive any oil or massage. All participants received similar treatment, other than application of SSO, and diet as per standard management protocol of the hospital. The study participants received care from dedicated study physicians and nurses who followed standard operating procedures for the study to give similar follow-up care to the study participants for both groups. Every episode of follow-up along with the medication, amount and frequency of diets were recorded in a chart for every participant in a similar manner for both treatment groups. Moreover, the participants of intervention and treatment arms stayed together in the same ward and received care from same staff at the same times. Thus, care for all patients was the same as per hospital policy, except the only difference was that the intervention arm participants received topical emollient SSO therapy three times daily during the study period.

### Outcome measures

The primary outcome was the day 10 rate of weight gain (g/kg body weight per day) based on nude weight of each participant taken at baseline and every morning for 10 days using a calibrated digital weighing scale (SECA, Model 2781321009, Germany; precision: 1 g). Secondary outcomes included: 1) rate of reduction in nosocomial infections (based on children with at least one infection), diagnosed clinically as per the hospital’s management guidelines [25] and identified as the appearance of any new sign of infection during the hospital stay that was not present at admission or not present during the preceding 48 hours of hospital stay; 2) mean time (days) to recovery from the acute phase based on improvement in mental state, normalisation of vital signs, re-establishment of oral feeding, and resolution of all acute illnesses; 3) changes in skin condition score at day 10 where an overall score of zero represents normal skin condition and the score increases with deterioration of skin condition and the proportion of body surface area involved (see Table S2 in the **Online Supplementary Document**; the score was adapted from the scoring system developed by Lane et al [26] as a measure of skin barrier integrity); 4) reduction in TEWL at day 10, a sensitive measure of skin barrier function [27]; and, 5) changes in C-reactive protein (CRP) levels at day 10, as an indicator of systemic inflammation [28]. Changes in levels of serum fatty acids and inflammatory cytokines, and in the composition of the skin and stool microbiome were also determined and are reported separately. To measure time to discharge from the acute phase and the presence of nosocomial infection, study physicians assessed and recorded clinical data on the participants using standardised case record forms. Clinical skin changes used to calculate skin condition scores were ascertained every 2 days by study physicians. TEWL was measured every 2 days with a MDD 4 Tewameter (Courage+Khazaka Electronic GmbH, Germany) after skin adaptation in a designated room for about 30 minutes at controlled temperature (20°C ± 1°C) and humidity (50% ± 10%). For blood biochemistry (eg, CRP), blood samples were taken on enrollment and again after completion of 10 days of intervention and were analysed at the icddr,b clinical laboratory. Other biochemical tests including serum electrolytes, calcium, magnesium and creatinine; hematological tests (eg, hemoglobin); and microbiological tests (eg, blood, stool and urine culture) were done at the icddr,b clinical laboratories at enrollment as a part of hospital standard-of-care. A pre-tested case record form was used to capture data in written form which were then transcribed to appropriate statistical software.

All adverse events (AEs) and severe adverse events (SAEs) were recorded and reported through established processes at icddr,b. Unanticipated SAEs that were likely due to emollient application (such as the child slipping off the caregiver’s lap resulting in an injury, severe hypersensitivity reaction due to the emollient, accidental choking or ingestion of emollient) were reported to the DSMB within 24 hours of the event. However, AEs including nosocomial infection, sepsis, septic shock or any other anticipated consequence of the ongoing illness were not reported to the DSMB as per the instruction of the IRB, although all AEs and SAEs were recorded on the case record form of each participant.

### Statistical analysis

Sample size calculation was based on the findings of a recently completed study of nutritional rehabilitation of children with SAM at icddr,b (unpublished data, sayeeda@icddrb.org, personal communication). The mean weight gain was comparable in children of mean age 12.6 (± 4.9) months who were given a ‘khichuri’ and ‘halwa’ diet – the standard-of-care at icddr,b hospital during nutritional rehabilitation therapy for children aged >6 months – vs children who received Ready-to-Use Therapeutic Foods like ‘Plumpy'Nut®’ (Nutriset, Malaunay, France) [29]. Children in our study received the former diet, and non breast-fed children aged 2 to <6 months in both groups received milk-based formula according to hospital evidence-based guidelines [24]. The study was therefore designed to detect a 2.0 g/kg/d difference in the rate of weight gain at day 10 between participants receiving emollient therapy vs participants receiving standard of care. Assuming a significance level of 0.05 and a 2-sided, 2-sample, unequal-variance *t* test with 80% power, and a 16% attrition rate, 100 subjects per arm were required to detect this difference. Out of the total sample size of 200 participants pre-specified to be enrolled, two-thirds (n = 133) would be in the 6 to 24 months age stratum and one-third (n = 67) would be in the 2 to <6 months age stratum.

Statistical analysis was performed at both icddr,b (using STATA, StataCorp, USA, version 13.0) and at GlaxoSmithKline, UK (using SAS, SAS Institute Inc., Cary, NC, USA, version 9.4) (see Appendix S1 in the **Online Supplementary Document** for a detailed description of the predetermined analysis plan) and validated by comparison of results. Any differences in results were resolved through discussions by analysts from icddr,b and GSK and examination of code to identify potential discrepancies. Statistical analysis was performed on data from all randomised patients in the study on an intention-to-treat basis. Data from children who were withdrawn because of failure to respond to the usual management of SAM, or voluntary dropouts, were included in the analysis up to the time of withdrawal. Summary statistics, including the minimum, maximum, median, mean, standard deviation (SD) and 95% confidence interval (CI), were calculated for all outcomes except time-to-discharge from the acute phase which was summarized through Kaplan-Meier analysis. A mixed model repeated measures (MMRM) was fitted to the weight, TEWL and skin condition scores with baseline value, age in months, calorie intake per day per kg (calculated by dividing the mean calorie intake by baseline weight and for weight only), mean breast feeding frequency (for weight only), treatment, day, sex, baseline value × day (× denotes an interaction term), treatment × day, treatment × age category, treatment × age category × day (age category as per the randomisation categories) as fixed effects and subject as the random effect. For time to discharge from the acute phase, a Cox proportional hazards model was fit with baseline weight-for-length z-score, age in months, treatment, and sex fit as fixed effects. Suspected nosocomial infections were modelled based on children with at least one infection using logistic regression with baseline weight-for-length z-score, age in months, treatment, and sex fit as fixed effects. All the endpoints analyses were also split by age group. For the statistical analysis, the same model was fit for the overall population and the calculation of the adjusted means modified based on the age category of interest.

### Patient and public involvement

No patients were involved in the design process of this study, setting the research question or the outcome measures, nor were they involved in the analysis, interpretation, and writing of the results. Our findings from the trial will be shared with all participants, who will be provided with a lay abstract of our study and access to the full manuscript.

## RESULTS

### Subjects

**Figure 1:**
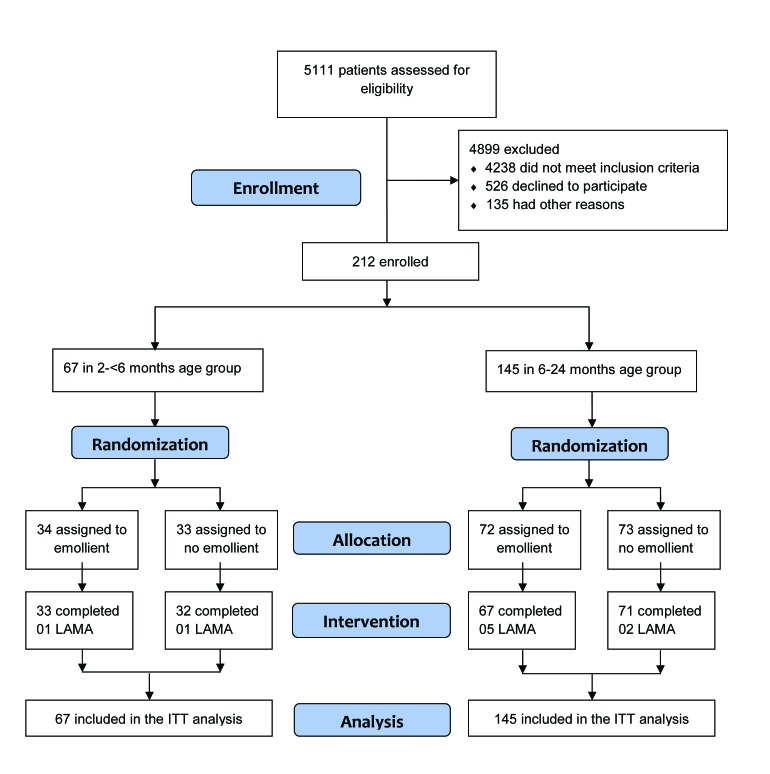
CONSORT flow diagram of the study. LAMA – left against medical advice; ITT – intention-to-treat.

Between January 2016 to December 2017, 5111 individuals were assessed for eligibility (**Figure 1**). Due to low enrollment of participants in the younger age stratum during the study period, we extended our enrollment period (after approval from the IRB) in order to reach the original sample size estimate. During that time, we also continued enrollment in the older age stratum beyond our initial target sample size. Thus, the final sample was 212, 67 in the 2 to <6 months age stratum and 145 in the 6 to 24 months age stratum (**Figure 1**). In the 6 to 24 months age stratum, 72 participants were enrolled in the emollient therapy arm and 5 participants left the hospital against medical advice before completion of the 10-day study period. Seventy-three participants were enrolled in the control (standard of care, no emollient therapy) arm and 71 completed the 10-day study. In the 2 to <6 months age stratum, 34 and 33 participants were enrolled in the emollient and control arms, respectively, and among them, 33 and 32 subjects completed the study. No participant died or developed any SAE during the study.

Baseline demographic and clinical characteristics were comparable between the treatment and control groups in the two age strata, with the exception of the sex distribution of participants in the younger age group, in which more male participants were enrolled in the treatment than the control arm (85% vs 39%, respectively) (**Table 1**).

**Table 1:** Baseline characteristics by treatment group for children ages 2 to <6 months and 6 to 24 months*

Variables	2 to <6 months		6 to 24 months	
**Emollient therapy (n = 34)**	**No emollient (n = 33)**	**Emollient therapy (n = 72)**	**No emollient (n = 73)**
Age, months	4.07 ± 1.14	4.05 ± 1.28	11.97 ± 4.12	12.81 ± 4.33
Male sex (%)	29 (85%)	13 (39%)	49 (68%)	48 (66%)
Weight, kg	4.53 ± 0.87	4.17 ± 0.67	6.02 ± 0.80	6.34 ± 0.85
Length, cm	60.22 ± 4.04	58.67 ± 3.10	68.73 ± 4.55	70.53 ± 4.78
Weight-for-length z-score	-3.59 ± 0.40	-3.46 ± 0.44	-3.52 ± 0.38	-3.50 ± 0.49
Mid-upper arm circumference, mm	112.82 ± 10.72	109.58 ± 7.24	119.75 ± 7.32	122.77 ± 7.47
Presence of oedema, n (%)	3 (9%)	1 (3%)	2 (3%)	1 (1%)
Immunization status up-to-date, n (%)	20 (59%)	16 (48%)	47 (65%)	54 (74%)
Duration of exclusive breastfeeding, months	1.03 ± 1.30	0.56 ± 0.73	2.28 ± 2.45	2.73 ± 2.30
Duration of diarrhea, days	4.41 ± 2.35	4.33 ± 1.71	4.25 ± 2.54	4.58 ± 2.34
Duration of fever, days	1.32 ± 2.29	0.85 ± 1.00	1.33 ± 2.15	1.00 ± 1.20
Haemoglobin, gm/dl	10.31 ± 1.29	10.58 ± 2.47	10.23 ± 1.81	10.68 ± 1.6
Total white blood cell count, per cm^3^	14446.76 ± 6374.54	14949.06 ± 4585.70	14951.04 ± 7403.5	14378.26 ± 4747.61
Total platelet count, per cm^3^	521264.71 ± 181686.09	508181.82 ± 219723.47	473486.11 ± 185032.70	455684.93 ± 139247.77
Serum sodium, mmol/L	136.82 ± 10.12	136.32 ± 6.84	134.97 ± 8.02	134.32 ± 4.48
Serum potassium, mmol/L	3.88 ± 0.77	4.09 ± 0.96	3.63 ± 0.75	3.84 ± 0.82
Serum creatinine, μmol/L	26.76 ± 17.01	26.38 ± 14.66	21.68 ± 5.82	22.30 ± 5.92
Serum calcium, mmol/L	2.36 ± 0.19	2.38 ± 0.18	2.38 ± 0.17	2.43 ± 0.12
Growth in urine culture, n (%)	21 (62%)	16 (48%)	25/70 (36%)	22/71 (31%)
Growth in stool culture, n (%)	9 (26%)	11 (33%)	26 (36%)	17 (23%)
Growth in blood culture, n (%)	1 (3%)	2 (6%)	9 (12%)	6 (8%)
Chest x-ray, pneumonia, n (%)	1 (3%)	2 (6%)	8/71 (11%)	3 (4%)

*All values are mean ± standard deviation unless stated otherwise.

### Weight gain

**Figure 2:**
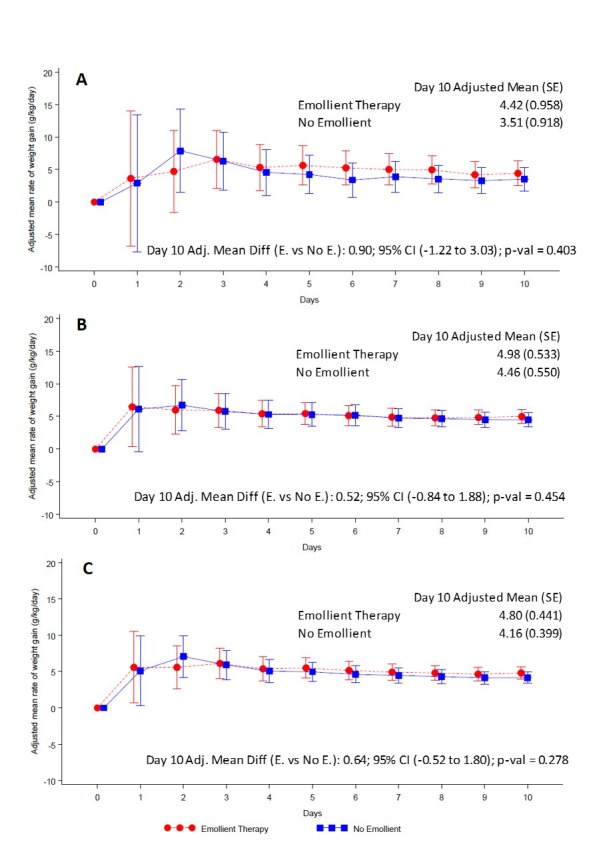
Adjusted mean (95% CI) rate of daily weight gain by treatment group. **Panel A.** Children aged 2 to <6 months. **Panel B.** Children aged 6 to 24 months. **Panel C.** All children. Red circles and line represent the emollient group and blue squares and line represent the control group. The analysis method was Mixed-Model Repeated Measures adjusted for treatment, baseline weight, day, age, sex, mean food intake per day per kg, mean breast feeding frequency, baseline weight × day, treatment × day, treatment × age category, and treatment × age category × day.

At the end of the 10-day study period, the adjusted rate of weight gain was 4.80 (standard error (SE) 0.44) g/kg/d in patients in the emollient arm and 4.16 (SE = 0.40) g/kg/d in the control arm (**Figure 2**, Panel C, **Table 2**). The difference in daily weight gain between the emollient and control groups was not significant [adjusted mean difference (AMD) = 0.64 g/kg/d, 95% CI = -0.52 to 1.80, *P* = 0.278) (**Figure 2**, Panel C). The difference in rate of weight gain between the emollient and control patients was greater in the 2 to <6 months age stratum (AMD = 0.90, 95% CI = -1.22 to 3.03) (**Figure 2**, Panel A) than in the 6 to 24 months age stratum (AMD = 0.52, 95% CI = 0.84 to 1.88) (**Figure 2**, Panel B), however, these differences were not statistically significant.

**Table 2:** Weight gain from baseline to day 10 of treatment for children ages 2 to <6 months and 6 to 24 months, by treatment group*

Agee group	Emollient therapy (N = 106)		No emollient (N = 106)		Rate of weight gain (g/kg/d)
	**Baseline**	**Day 10**	**Baseline**	**Day 10**	Adjusted difference* (95% CI) at Day 10
	mean (SD)	mean (SD)	mean (SD)	mean (SD)	Emollient therapy minus No emollient
**All**	5.6 (1.09)	5.8 (1.19)	5.7 (1.27)	5.9 (1.35)	0.64 (-0.52, 1.80)
**2 to <6 months**	4.5 (0.85)	4.6 (0.82)	4.2 (0.67)	4.3 (0.67)	0.90 (-1.22, 3.03)
**6 to 24 months**	6.0 (0.80)	6.4 (0.90)	6.3 (0.85)	6.6 (0.91)	0.52 (-0.84, 1.88)

CI – confidence interval

*Mixed-model repeated measures adjusted for treatment, baseline weight, day, age, sex, mean food intake per day per kg, mean breast feeding frequency, baseline weight × day, treatment × day, treatment × age category and treatment × age category × day.

### Nosocomial infections

The most common sign of nosocomial infection (based on count data, **Table 3**) during the study period was fever (n = 56), which was not classified further due to lack of data from blood cultures, followed by pneumonia (n = 9). The cumulative rate (combining the incidence of all suspected nosocomial infections for each individual) based on children with at least one infection was similar in the treatment and control groups for all patients together, but in the older age stratum, it was significantly lower in the emollient than the control group (adjusted odds ratio: 0.41, 95% CI = 0.19 to 0.85; *P* = 0.017) (**Table 4**).

**Table 3:** Total number of nosocomial infections by treatment group and age category

Nosocomial infection type	Total number of infections	2 to <6 months		6 to 24 months	
**Emollient therapy (n = 34)**	**No emollient (n = 33)**	**Emollient therapy (n = 72)**	**No emollient (n = 73)**
**Fever**	56	5	3	17	31
**Pneumonia**	9	1	0	5	3
**Urinary tract infection**	1	0	0	1	0
**Sepsis**	2	1	0	1	0

**Table 4:** Children with at least one nosocomial infection by treatment group and age category

Age group	Number of children with at least one nosocomial infection*, n (%)		Adjusted odds ratio† (95% confidence interval)	*P*-value
	**Emollient therapy**	**No emollient**
**All (n = 212)**	25 (24%)	36 (34%)	0.73 (0.30, 1.79)	0.499
**2 to <6 months (n = 67)**	6 (18%)	3 (9%)	1.31 (0.26, 6.63)	0.74
**6 to 24 months (n = 145)**	19 (26%)	33 (45%)	0.41 (0.19, 0.85)	0.017

*Including nosocomial fever, nosocomial pneumonia, nosocomial urinary tract infection and nosocomial sepsis

†Logistic regression adjusted for treatment, baseline weight-for-length Z score, age, sex, treatment × age category.

### Time to recovery from acute illness

**Figure 3:**
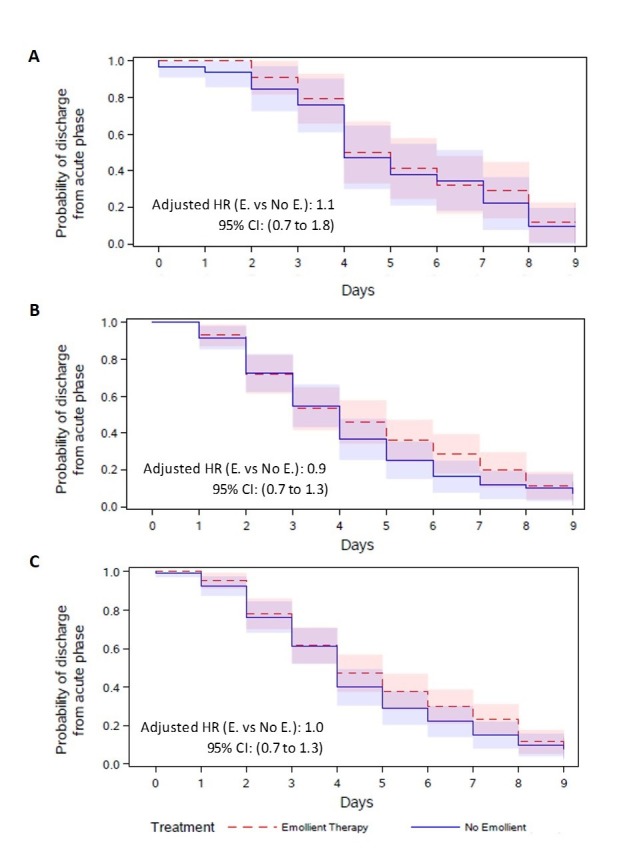
Kaplan-Meier plot of time (days) to discharge from the acute phase, by treatment group. **Panel A.** Children aged 2 to <6 months. **Panel B.** Children aged 6 to 24 months. **Panel C.** For all children. Red dot lines represent the emollient group and blue lines represent the control group. Hazard ratios were calculated from a cox proportional hazards model adjusted for treatment, baseline weight-for-length z-score, age and sex.

There was no difference in the time-to-discharge from the acute phase (adjusted hazard ratio 1.0 [95% CI = 0.7 to 1.3]), with a median discharge time of 4 days in both the emollient and control groups. The differences were not significant in either age strata (**Figure 3**).

### Skin condition and water loss

**Figure 4:**
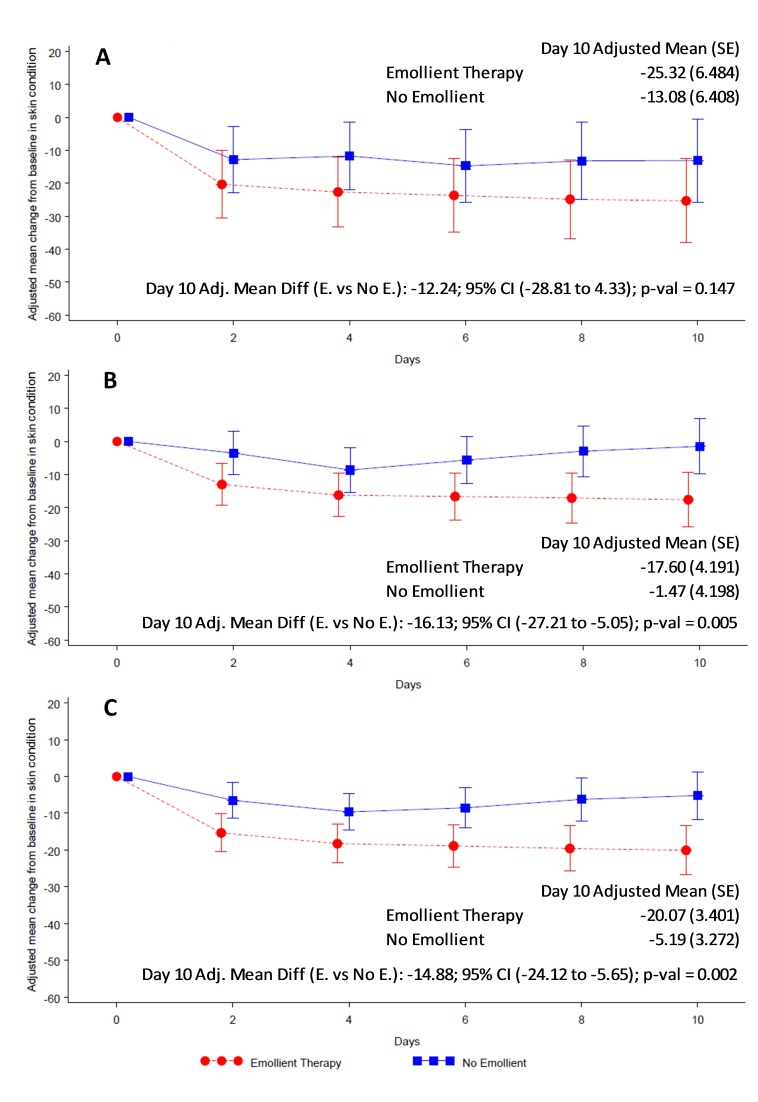
Adjusted mean (95% CI) change from baseline in skin condition scores by treatment group. **Panel A.** Children aged 2 to <6 months. **Panel B.** Children aged 6 to 24 months. Panel C. For all children. Red circles and line represent the emollient group and blue squares and line represent the control group. The analysis method was Mixed-Model Repeated Measures adjusted for treatment, baseline skin appearance score, day, age, sex, baseline skin appearance score × day, treatment × day, treatment × age category and treatment × age category × day.

**Figure 5:**
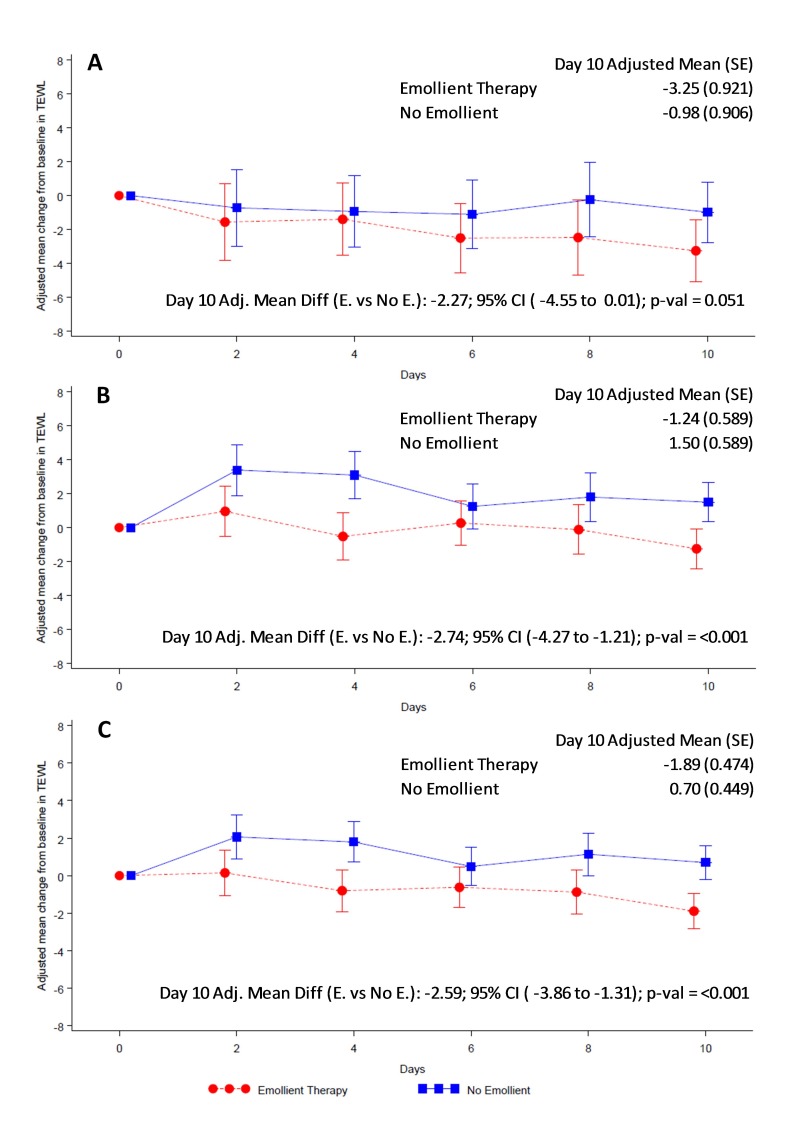
Adjusted mean (95% CI) change from baseline TEWL by treatment group. **Panel A.** Children aged 2 to <6 months. **Panel B.** Children aged 6 to 24 months. **Panel C.** For all children. Red circles and line represent the emollient group and blue squares and line represent the control group. The analysis method was Mixed-Model Repeated Measures adjusted for treatment, baseline TEWL, day, age, sex, baseline TEWL × day, treatment × day, treatment × age category, and treatment × age category × day.

Overall skin condition score was reduced (ie, skin condition improved) in both study arms from baseline to end of treatment (**Figure 4**). Reduction in skin score was significantly greater in the emollient arm compared to the control arm in all participants (AMD = -14.88, 95% CI = -24.12 to -5.65; *P* = 0.002) (**Figure 4**, Panel C) and in the older age stratum (AMD = -16.13, 95% CI = -27.21 to -5.05; *P* = 0.005) (**Figure 4**, Panel B). Reduction in skin score in the younger age group (AMD = -12.24, 95% CI = -28.81 to 4.33; *P* = 0.147) (**Figure 4**, Panel A) did not reach statistical significance. TEWL was significantly reduced in the emollient-treated participants compared to the control subjects in all participants (AMD = -2.59, 95% CI = -3.86 to -1.31; *P* < 0.001) and in both age strata (**Figure 5**).

### C-reactive protein

Changes in CRP levels from baseline (mean = 1.12, SD = 1.66 and mean = 1.29, SD = 3.14) to end of treatment (day 10) (mean = 0.27, SD = 0.21 and mean = 0.38, SD = 0.66) were similar in the emollient and control groups, respectively, for all patients, but in the younger age stratum the reduction was significantly higher in the patients treated with emollient compared to the control (median: -0.28 in emollient vs 0.00 in control, *P* = 0.019) (Table S3 in the **Online Supplementary Document**).

## DISCUSSION

Emollient therapy had many of the benefits we hypothesised, including improved skin condition (based on visual inspection for signs of compromised skin integrity) and skin barrier function (based on measurement of the rate of water loss through the skin) in children 2 to 24 months of age, and reduction in nosocomial infections (primarily fever) and in inflammatory markers (eg, CRP) in subgroups (by age) of treated children. While treatment with emollient therapy showed a trend toward increased weight gain, especially in the younger patients, this effect did not reach statistical significance, and time to clinical recovery from the acute phase of illness was not measurably impacted. While the benefits of emollient therapy on the skin barrier could be seen rapidly, within 2 days of treatment (the first post baseline measurement) – consistent with findings in animal models of the human skin barrier and in preterm infants with compromised skin barrier function [11,21,26] – and there were indications of benefits on health and growth within the 10-day time frame for the study, this may have been an insufficient period of time to fully realise the benefits of emollient therapy in young children with SAM. Most children were expected to be discharged from the acute phase to the rehabilitation phase after 3 days. However, the median number of days until transition to the acute phase was four in both treatment groups. Given that it takes additional time for the children’s weight to stabilise, the actual time in the study for them to gain weight was much less than 10 days, which was probably too short. Thus, future studies should incorporate a longer intervention period.

Infections are a principal cause of death among children with SAM. Importantly, we found that among the older children (6-24 months age stratum) in the emollient group, the odds of developing nosocomial infection was reduced by 59% compared to children in the control group. Consistent with trials of emollient therapy in preterm newborn infants with compromised skin barrier function – showing on average 50% reductions in the rate of nosocomial infection [11], proportionate to improvements in skin condition [30] – these results suggest that the skin may be a more important portal of entry for infections in children with SAM than previously recognised. This finding is consistent with the realisation that skin barrier integrity and resident microbiota distribution and behaviour are strongly interrelated [30]. Changes in skin and gut microbiota in the course of emollient therapy will be reported separately.

In paediatric clinical practice, C-reactive protein (CRP) is one of the most useful markers for monitoring patient response to therapy for infectious illnesses [31]. The level of this acute phase reactant reduces over time in response with therapy. We found that reductions in CRP levels were higher in the 2 to <6 month-old infants treated with emollient compared to the control infants. This may have been due to reduced skin inflammation associated with improved skin barrier function, as well as reduced cutaneous penetration of pro-inflammatory microbial products.

Although the study failed to demonstrate that emollient therapy increased the rate of weight gain by 2g/kg body weight per day, as originally hypothesised, trends for improved weight gain compared to controls were found. The rate of weight gain was also slightly higher among the younger children (2 to <6 months) compared to the older children (6 to 24 months). Prior studies have shown significantly greater weight gain (about 1.5 g/kg/d) over the neonatal period in preterm infants treated with emollient therapy compared to controls [12]. However, the limited duration of treatment for 10 days in combination with a short median time to discharge from the acute phase of 4 days may have been insufficient to discern measurable changes in rate of weight gain in this study.

There are several potential pathways whereby emollient treatment in children with SAM may result in improvements in health. SSO applications in preterm infants preserve skin integrity and protect the skin from injury [30]. Topical applications of emollients such as SSO help keep the skin hydrated and flexible, making it less likely to dry out and crack as skin flexes, especially around the infants’ joints [32], which is also reflected by the improved skin condition observed for the emollient group. In addition, absorption and metabolism of EFAs from SSO may result in acceleration and augmentation of skin barrier formation and function [33]. The major lipid constituent of high-linoleate SSO is linoleic acid (18:2 n-6) [22], which has been shown to have a direct role in epidermal permeability barrier repair and augmentation of skin barrier function in states of nutritional deficiency [6,20,22,33,34]. Keratinocytes – the primary cell type comprising skin – have specific receptors that bind linoleic acid and facilitate its uptake. Linoleic acid converts to arachidonic acid (20:4 n-6) and is a precursor to prostaglandin E2 (PGE2), a known modulator of cutaneous inflammation [35]. Linoleate has also been shown to be among the most potent activators of peroxisome proliferator-activated receptor-α (PPARα), and its application to fetal rat skin explants at physiologic concentrations has been shown to accelerate epidermal barrier development through stimulating PPAPα and up-regulating genes controlling skin development [36]. Linoleic acid also up-regulates the production of antimicrobial peptides and induces keratinocyte differentiation (ie, expression of involucrin and transglutaminase), thus promoting skin maturation [23]. Although EFAs are not synthesised in situ, when applied exogenously, they exert multiple actions locally and systemically that likely contribute to the beneficial effects seen with SSO applications in children with SAM.

Vegetable oils are readily available, inexpensive, and hence, commonly used as topical therapy in children in many developing countries, particularly in South Asia and the Mediterranean region; use has also been documented in sub-Saharan Africa [13,14,27,37-39]. In Bangladesh, oil massage is culturally well accepted, typically started in the first few days after birth and continued for months to years of a child’s life, based on the perception that this practice will help keep the skin smooth, strengthen the bones, and protect from cough and cold [39]. Oils are also applied to a variety of skin conditions with the aim to accelerate healing [39]. However, the most commonly used oil – mustard oil – is potentially toxic to skin and lacks a favorable fatty acid profile [33]. Thus, taken together, available data suggests that through provision of both essential and non-essential fatty acids to the skin and to the systemic circulation following transcutaneous absorption, topical applications of SSO may provide a culturally acceptable, affordable [16] means of improving epidermal barrier function and health of young children in developing countries with SAM.

### Comparison with other studies

This is the first trial of skin barrier therapy as an adjunctive treatment for children with SAM. Results reported here suggest that skin barrier repair therapy may provide an alternative, adjunctive approach to rehabilitation of children with SAM. No data are available, and testing of this approach may also be warranted in children with moderate acute malnutrition.

### Strength and weaknesses of this study

The study had several limitations. Due to the hospital setting, it was challenging to keep the caregivers at the hospital for 10 days to complete the treatment, especially during the rehabilitation phase of SAM, when the acute, life-threatening signs of the illness had subsided. Nevertheless, the majority of subjects completed the planned 10-day study, but this was likely an insufficient period of time in the rehabilitation phase (average 6 days) for manifestation of the added benefits of topical SSO therapy beyond that achieved through standard-of-care for SAM, which includes intensive feeding by mouth. All eligible participants were enrolled – boys and girls – at the hospital. However, the overall percentage of male children was significantly higher than females in the 2 to <6-month age stratum. This was due to the higher proportion of male children who were brought to the hospital during the trial period, possibly reflecting better care seeking behavior of the caregivers for their male children compared to female children in this population [40]. Future trials in this setting should consider stratifying the randomisation by sex as well as age. We diagnosed nosocomial infection on the basis of clinical signs and symptoms. Due to resource limitations, we were unable to perform microbiological culture in all cases of suspected serious infection. Thus, the efficacy of emollient therapy in preventing infections in children with SAM should be confirmed through use of definitive detection techniques such as blood culture. Moreover, although a higher number of nosocomial infections was found in the treatment arm compared to control arm in the younger age group, the number was very low and it was not statistically significant. Future studies should be designed to test for statistical significance in nosocomial infections. In the study there was high acceptability of emollient treatment. Most caregivers implored the study staff to provide them with SSO to enable them to continue treatment of their child at home after the completion of the trial.

## CONCLUSIONS

We found that topical emollient therapy with SSO was safe, highly acceptable and had positive health impacts in children with SAM when applied as a brief adjunctive therapy alongside the current standard-of-care. Emollient therapy improved skin condition and barrier function, reduced the risk of nosocomial infection, and showed a trend for increasing the rate of weight gain. However, further research in other populations with longer treatment duration and follow-up, and microbiological diagnosis of infections is needed to strengthen the findings of the study.

## Additional material

Online Supplementary Document
