# Supplementary material for: Topical emollient therapy in the management of severe acute malnutrition in children under two: A randomized controlled clinical trial in Bangladesh
Source: J Glob Health. 2020 May 15;10(1):010414. doi: 10.7189/jogh.10.010414 (PMC7243074; doi:10.7189/jogh.10.010414)
Supplement: Online Supplementary Document [file jogh-10-010414-s001.pdf]

**Title:**	Reporting and Analysis Plan for an Exploratory Study to Evaluate the Use of Topical Emollient Therapy in the Management of Severe Acute Malnutrition: A Randomised Controlled Clinical Trial in Bangladesh

Description: This document provides the reporting and analysis plan for this exploratory randomised controlled open-label clinical trial investigating topical emollient therapy in the management of severe acute malnutrition in children aged 2 – 24 months in Bangladesh

ABBREVIATIONS

ANCOVA	Analysis of Covariance
ASOM	Acute Suppurative Otitis Media
CI	Confidence Interval
CRF	Case Report Form
CRP	C-Reactive Protein
CSOM	Chronic Suppurative Otitis Media
FA	Fatty acid
GSK	GlaxoSmithKline
icddr,b	International Centre for Diarrhoeal Disease Research, Bangladesh
ITT	Intent-to-Treat
MMRM	Mixed Model Repeated Measures
R&D	Research and Development
RAP	Reporting and Analysis Plan
SAM	Severe Acute Malnutrition
SAS	Statistical Analysis Software
SD	Standard Deviation
SPSS	Statistical Package for the Social Sciences
SSO	Sunflower Seed Oil
TEWL	Transepidermal Water Loss
UTI	Urinary Tract Infection

1. OBJECTIVE(S) AND ENDPOINT(S)

1.1. Objective(s)

- To evaluate the benefit of topical applications of sunflower seed oil (SSO) to children aged 2-24 months with SAM

1.2. Endpoint(s)

This is an exploratory open-label study to evaluate potential benefits of topical application of (SSO in SAM. There is little or no historical data available on efficacy endpoints, therefore an interim analysis was planned when 100 evaluable subjects have completed the study. The results from the interim analysis will help identify key/primary efficacy endpoints. Therefore, the list of endpoints and analysis plan may be updated after the interim analysis.

The endpoints which will be investigated in the analysis are:

<u>Primary Endpoints</u>

- Weight gain (g/kg/day)

<u>Secondary Endpoints</u>

- Time (days) to discharge from acute phase
- Rate of suspected nosocomial infections (nosocomial infection will be diagnosed clinically as per hospital's management guidelines where it has been mentioned that any new sign of infection during hospital stay that was not present at admission will be considered as nosocomial infection. Following the interim analysis, the list of nosocomial infections was restricted to pneumonia, fever, UTI and sepsis.)
- Change from baseline in transepidermal water loss (TEWL) at Day 10
- Change from baseline in plasma fatty acid levels at Day 10*
- Change from baseline in c-reactive protein (CRP) at Day 10
- Change from baseline in pro-inflammatory cytokine parameters at Day 10

- Clinical improvement of visual skin condition:
  - Change from baseline in skin redness at Day 10
  - Change from baseline in skin condition score at Day 10

- Change from baseline in the skin and gut microbiome data at Day 10*

* Change from baseline in plasma fatty acid levels at day 10 will not be included in the interim analysis as the results will not be available until the end of the study. The analysis of plasma fatty acid levels will be included in the final study report. However, this analysis is outside the scope of this RAP.

* Change in the skin and gut microbiome data will be collected for a sub-sample of subjects in the study as described in the protocol amendment. This analysis will be performed at the end of the study by an external expert at Stanford University. The metabolomic data will be analysed by the CRO (Metabolon) and additional support will be provided by the GSK subsidiary company Cellzome. As previously indicated, the analysis of skin and gut microbiome data is outside the scope of this RAP.

2. STUDY DESIGN

Please refer to the protocol for details on the study design and sample size.

3. PLANNED ANALYSES

An interim analysis was planned when approximately 100 evaluable subjects have completed the study. For further details of the planned interim analysis, refer to the RAP version 1 dated 09-Jun-2016.

A final analysis will be conducted when all subjects have completed the study and all data have been entered, checked and cleaned.

4. ANALYSIS POPULATIONS

The following populations will be defined:

Safety population: The safety population will comprise all randomised subjects who start the SAM Routine Standard of care (with or without topical application of SSO) and will be used for the analysis of safety data. The data will be analysed according to the actual treatment received if this differs from the treatment to which the subject was randomised. If subjects receive treatment differing from that assigned by the randomisation schedule (for either a portion of or the entire time on study), they will be

analysed based on the treatment taken for the majority (>50%) of their time on study treatment.

Intent-to-Treat (ITT) population: This will consist of all randomised subjects from whom at least baseline and at least one post baseline measure have been obtained and will be used for the analysis of efficacy data. The data will be analysed according to the treatment to which the subject was randomised.

5. TREATMENT COMPARISONS

5.1. Data Display Treatment and Other Sub-group Descriptors

The descriptors as detailed below will be used to denote the study treatment and will be used in the data displays:

- Emollient Therapy
- No Emollient

6. GENERAL CONSIDERATIONS FOR DATA ANALYSES

All programming will be performed using - SAS (version 9.4 or later), SPSS or STATA. The analysis will be carried out in collaboration with icddr,b, and Stanford University. Further analysis of the SCORDoK score may be carried out by the Denmark SCORDoK team. The metabolomic data will be analyzed by the CRO assaying the samples (Metabolon) with input from representatives from the R&D Platform Technology & Science group within the GSK subsidiary company Cellzome All analyses will be deemed exploratory.

6.1. Strata and Covariates

Randomisation was planned to be stratified by age at baseline in two strata: 2-<6 months and 6-24 months in a 1:2 ratio. However, as noted in the amended protocol, additional subjects may be enrolled in the older subgroup.

The two age group strata (2-<6 months and 6-24 months) will be the categories used in all tables, figures and listings split by age group unless stated otherwise.

The statistical analysis will be adjusted for age at baseline (as a continuous covariate), sex and a measure of the parameter or severity at baseline as specified in the table below. In addition, the analysis of weight gain will include average calorie intake divided by baseline weight (calorie intake per day per kg).

Patients will be categorised as “Oedematous” if they are recorded on the CRF as having oedema at any time during the study. Otherwise they will be categorised as “Non-oedematous”. For each patient, the daily calorie intake (total energy per day) is collected on the CRF. The average of these values divided by baseline weight will be used as the calorie intake covariate.

If an inadequate number of subjects is observed in one of the categories for sex or oedematous status then the covariates may be removed from the models.

In addition to the above, differences between the two treatment groups (emollient therapy versus no emollient) will be examined with respect to baseline characteristics prior to performing statistical analysis of the efficacy endpoints. If any differences are considered to be clinically important, exploratory subgroup analysis may be presented for the relevant endpoints.

Depending on the method of statistical analysis, covariates/factors specified below will be fitted in each model. If any terms are not significant then they may be dropped from the final analysis.

Table 1: Covariates/Factors to be included in statistical analyses

Method of analysis	Endpoints	Fixed effects terms			Random effects terms
		Continuous Covariates	Factors	Interactions	Covariates
ANCOVA	Fatty Acids	Baseline of parameter, age	Treatment, sex		
MMRM	Weight<br><br>TEWL<br><br>Skin redness<br><br>Skin Appearance	Baseline of parameter, age, calorie intake per day per kg[2], mean breast feeding frequency[2]	Treatment, day, sex, oedematous status	Baseline of parameter *day, treatment*day, treatment*age category, treatment*age category*day [3]	Subject (in the subject option of the repeated statement in proc mixed)
Cox proportional hazard model	Discharge from acute phase	Baseline weight-for-length Z score, age	Treatment, sex		

Logistic regression	Suspected nosocomial infections	Baseline weight-for-length Z score, age	Treatment, sex		

[1] Oedematous status and calorie intake was only to be included in the analysis of weight at interim. After interim, since there were only 4 subjects with oedema, the decision was taken to remove subjects with oedema from the statistical analysis. Oedematous status was therefore removed as a covariate.

[2] Based on further information from the data and from site the decision was taken to adjust for calorie intake per day per kg rather than calorie intake. Also, it was decided to use mean breast feeding frequency as a covariate for the weight analysis only

[3] The additional interactions of treatment*age category and treatment*age category*day was included after the review of interim analysis results to investigate the differences in endpoints between the two age categories. Note, the age categories are: 2-<6 months and 6-24 months.

[4] Note, the statistical analysis of endpoints related to the skin and gut microbiome data is not included here, because this analysis will be performed by experts in the field at Stanford University, California, USA. Also, the analysis of the metabolomic data will be performed by a CRO Metabolon with additional input from representatives of the GSK subsidiary company Cellzome.

6.2. Multiple Comparisons and Multiplicity

As this is an exploratory study, no adjustment will be made for multiple testing. When interpreting individual p-values for the endpoints, care will be taken to consider the results as part of the wider study results.

6.3. Missing Data

No imputation will be carried out for missing data or subjects who withdraw early. Data from children withdrawn because of failure to respond to the usual management of SAM, or voluntary dropouts will be included in the analysis up to the time of withdrawal. A supplementary analysis excluding the children withdrawn may be performed.

An exception to this is the Mean Breast Feeding Frequency variable for the Weight Gain analysis. In cases where there were missing values for all ten days, the mean value was imputed to be 0 rather than missing for modelling purposes.

7. DERIVED DATA

7.1. Baseline

For assessments that were collected on the daily monitoring chart, baseline is considered to be the subjects Day 1 assessment as collected on the CRF. For all other assessments, baseline is considered to be the subject's Day 0 assessment as collected on the CRF. If there are multiple assessments on Day 0 (or Day 1 for assessments from the daily monitoring chart) then the earliest will be taken as the baseline.

Change from baseline for post-baseline records will be calculated by subtracting the baseline value from the post-baseline value. If either the baseline or post-randomisation value is missing, the change from baseline is set to missing.

Percentage Change from Baseline will be calculated as

$$\frac{Visit\ Value - Baseline\ Value}{Baseline\ Value} x100$$

7.2. Study Day

As noted in section 8.1, study days have been recorded or labelled differently across different CRF forms. Study days are recorded from day 1 to day 11 on the daily monitoring chart, day 1 to day 10 on the Emollient record and Calorie intake CRF and from day 0 to day 10 in the other CRFs. Therefore, it is possible that the same date will be assigned a different study days across different domains. Following review of interim analysis results, in order to resolve this inconsistency or confusion in reporting, all study visits will be presented from Day 0 to Day 10. See Table 2 below for further clarification.

Table 2: Clarification of Study day to be used during final reporting

**Label to be used throughout the final study report**	**Day as recorded in the Daily Monitoring Chart (CRF/Dataset)**	**Day as recorded in the Emollient record and Calorie intake (CRFs/Datasets**
Day 0	Day 1	Day 1
Day 1	Day 2	Day 2
Day 2	Day 3	Day 3
Day 3	Day 4	Day 4
Day 4	Day 5	Day 5
Day 5	Day 6	Day 6

Day 6	Day 7	Day 7
Day 7	Day 8	Day 8
Day 8	Day 9	Day 9
Day 9	Day 10	Day 10
Day 10	Day 11	

Note: Label Day 0 is also referred to as baseline in this RAP.

Assessments that were collected three times a day on the daily monitoring chart will have the time of assessment included in the day description. For example, day 1 will have the following labels: Day 1 9AM, Day 1 5PM and Day 1 1AM. For variables with multiple assessments on a given study day, first available assessment value will be used for the analysis.

8. STUDY POPULATION

8.1. Disposition

The number and percentage of subjects who completed the study and withdrew prematurely from the study will be presented by treatment group for the Safety population. The table will also summarise the reason for premature withdrawal.

8.2. Demography

Demographic characteristics will be summarised by treatment group for the Safety Population. Summary statistics (number of subjects, mean, and standard deviation, median, minimum and maximum) will be presented for age (months), weight (kg) and height (cm) at Day 0. The number and percentage of subjects will be presented for sex and age group (2-<6 months, 6-24 months) at Day 0.

8.3. Baseline Characteristics

Summary statistics for the weight-for-length Z (WLZ) score, length-for-age Z (LAZ) score, weight-for-age Z (WAZ) score, mid upper arm circumference, mother's / caregiver's formal years of education, father's formal years of education and duration of exclusive breast feeding at Day 0 will be summarised by treatment group for the Safety population. The number and percentage of subjects will also be presented for, presence of oedema, breast feeding history (no breast feeding since birth, partial breast feeding or exclusive breast feeding), breast feeding status (still breast feeding, not still breast feeding), type of diarrhoea and presence of pneumonia, sepsis/septicaemia, meningitis/encephalitis, vitamin A deficiency, skin infection, ileus, ASOM/CSOM, enteric fever and other clinical diagnosis and visible wasting at Day 0.

For the interim analysis only, differences between the two treatment groups (emollient therapy versus no emollient) for each baseline characteristic will be examined. For continuous data a two sample t-test will be carried out to compare the two treatment groups. If the data is not normally distributed then a non-parametric Wilcoxon rank sum test may be used instead. For categorical data a chi-squared test will be used.

9. EXPLORATORY EFFICACY ANALYSES

All efficacy tables, listings and figures will use the ITT population unless specified otherwise.

9.1. Weight

<u>Derivations</u>

Weight is collected once every day (approximately at 9 am) and recorded on ‘daily monitoring chart’ for all participants. However, for intervention group only, weight is measured three times daily (approximately at 9 am, 5 pm and 11 pm) and recorded on ‘Emollient application record’ to measure the intervention material. The summaries and analysis of weight gain will be based on the daily 9AM measurements on ‘Daily monitoring chart’ only, however, all measurements will be considered in the derivation of baseline weight. Data from baseline up to and including day 10 (labelled as Day 1 to Day 11 on the daily monitoring chart CRF) will be included in the weight analysis as per the specification on the eCRF. At the time of interim and post interim, a footnote will be included to indicate this.

Weight measurements that were collected while a subject had detectable oedema will be excluded from the summaries and analysis. The earliest measurement in the daily monitoring chart where oedema status was recorded as 1 (no oedema) will be taken as the baseline weight. Following review of the interim analysis results, it was decided that all subjects with oedema would be excluded from the weight analysis only. Subjects with oedema would be summarised separately in the final clinical study report.

Weight will be summarised as a rate of weight gain (g/kg/day), as defined below:

Change from Baseline in Weight at Day X (g) = Weight at Day X (g) – Weight at Baseline (g)

Relative Change from Baseline = Change from Baseline in Weight at Day X (g)
____

in Weight at Day X (g/kg) — Weight at Baseline (kg)

$$\text{Rate of Weight Gain at Day X (g/kg/day)} = \frac{\text{Relative Change in Weight at Day X (g/kg)}}{\text{X}}$$

Note: 'X' refers to the study day label (see Table 2, page 10) and not the day reported on the daily monitoring chart

<u>Summary Statistics</u>

The following endpoints will be summarised by day (day 0 – day 10), presenting the number of subjects, mean (with standard deviation) and median, minimum and maximum for each treatment group. The summaries for change from baseline, relative change from baseline and rate of weight gain will also present summary statistics for the last day the subject was in the study.

- Weight (kg)
- Weight (kg) by oedematous status
- Change from baseline in weight (g)
- Change from baseline in weight (g) by oedematous status
- Relative change from baseline in weight (g/kg)
- Relative change from baseline in weight (g/kg) by oedematous status
- Rate of weight gain (g/kg/day)
- Rate of weight gain (g/kg/day) by oedematous status

Relative change from baseline will also be plotted over time. Day will be presented on the x-axis and the relative change from baseline will be presented on the y-axis. The mean at each day will be presented with an associated 95% confidence interval (CI). The plot will be repeated for the rate of weight gain.

Additionally, the WAZ, WLZ and LAZ scores will be presented over time using simple descriptive statistics tables and graphical plots. Further exploratory statistical analyses may be performed for one or more Z scores if the review of data warrants so.

<u>Mixed model repeated measures analysis</u>

Rate of weight gain (g/kg/day) will be analysed using a mixed model repeated measures (MMRM) analysis with restricted maximum likelihood estimation and an unstructured variance-covariance matrix. Covariates/ factors will be included in the model as

described in Section 7.1**Error! Reference source not found.**. In the circumstance that there are convergence problems with the MMRM analysis, this will be explored. For example the SCORING=4 option could be used in the MIXED statement, which makes SAS use Fisher scoring for the first 4 iterations. If the convergence problem cannot be resolved then alternative covariance structures will be investigated.

The estimate of treatment differences for “Emollient Therapy – No Emollient” at each day will be displayed in the summary of statistical analysis together with the 95% CI and the associated p-value. Least Squares Means for each day will also be presented with the standard error and the number of subjects contributing to the Least Squares Means.

The least squares mean at each day will be plotted by treatment group. Day will be presented on the x-axis and the rate of weight gain will be presented on the y-axis. The mean at each day will be presented with an associated 95% CI.

If any of the covariates are statistically significant then post-hoc analyses may be conducted to investigate further.

The normality assumptions will be assessed by inspection of the following plots:

Histogram of marginal studentised residuals derived from the MMRM model

- Normal probability plot

Bayesian analysis

At the interim analysis stage, Bayesian methods of analysis will be used to estimate the posterior distribution for the treatment difference in rate of weight gain between emollient therapy and no emollient at day 10 for the ITT population.

The Bayesian analysis will be based on a non-informative prior, using the prior statement within the mixed procedure in SAS (with the default Jeffreys prior option). The posterior distribution will be obtained from 10,000 simulations however if the Monte Carlo standard error is less than 0.015 then the number of simulations may be increased.

A table showing the posterior probabilities of the difference in rate of weight gain between emollient therapy and no emollient at day 10 being greater than 0, 1, 2 and 4, and the 95% credible interval, will be produced. A figure of the posterior distribution will also be produced.

No further Bayesian analysis of the data at the end of the study is planned.

Further analysis

Non-linear modelling and/or non-parametric analysis may be considered if deemed appropriate.

Post interim analysis changes

After the interim analysis further investigation led to the inclusion of mean breast feeding frequency as an additional covariate. This was calculated as the mean value of available breast feeding frequency information and if all of this data was missing, the mean breast feeding frequency was imputed as missing.

Food intake was also modified as a covariate. Initially, the mean total food intake per day for a subject was taken to be a covariate but this was later changed to be calorie intake per day per kg as calculated by the following:

$$\text{Calorie Intake per day per kg} = \frac{\text{Mean Calorie Intake per Day}}{\text{Weight at Baseline (kg)}}$$

Data for calorie intake was only available from Day 1 to Day 10 and the resulting variables calculated accordingly

Additional interactions highlighted in Section 7.1 have been included to investigate the effect of age category (based on the randomized strata) on the weight analysis. The decision was made to obtain estimates and treatment differences by age category and all subjects through one model. The OM option in the LSMEANS statement of PROC MIXED was used to obtain separate margins for each level of the LS-means effect for the categorical variables, Sex and Age Category. Instead of using equal coefficient across the classification effects for standard LS-means, the OM option allows these coefficients to be proportional to those found in the observed dataset.

9.2. Time (days) to discharge from acute phase

Derivations

Discharge from the acute phase is based on improvement in mental state, normalisation of vital signs, re-establishment of oral feeding and resolution of all acute illnesses.

The CRF collects information on whether a patient is ready for discharge from the acute phase (Y/N) daily. Time (days) will be derived as the number of days between baseline and the first occurrence where 'Ready for discharge from acute phase' was recorded as Yes. Baseline will be taken as the earliest measurement recorded in the daily monitoring chart. Day will be derived relative to the baseline.

If a subject withdrew or died before being ready for discharge from the acute phase then they will be censored at the time they left the study. Patients who completed Day 10 before being ready for discharge will be followed up by the site until they withdraw or are ready for discharge.

Kaplan Meier

A Kaplan-Meier plot including 95% CIs and the number of subjects at risk at each day will be presented.

Cox Proportional Hazards

The time to discharge from acute phase will be compared between treatment groups using a Cox's Proportional Hazards model adjusting for the covariates specified in Section 7.1.

The time to discharge from acute phase will be presented in quartiles for each group. The hazard ratio, 95% CI and associated p-value will also be presented.

Residual plots from the Cox proportional hazard model and the Kaplan-Meier plot will be used to visually check the assumption of proportional hazards is fair. If the proportional hazard assumption is invalid, then other methods of analysis may be considered which do not make this assumption, for example a Weibull model may be fitted to the data.

9.3. Rate of suspected nosocomial infections

Derivations

Any new sign of infection during hospital stay that was not present at admission or not present for last 48 hours of hospital stay will be considered as nosocomial infection. Occurrence of the following four suspected nosocomial infections, pneumonia, fever, UTI and sepsis, is collected on the CRF. Subjects will be considered as having had a suspected nosocomial infection if they have at least one of these four infections where symptoms started during the 10-day treatment period. Pneumonia, fever, UTI and sepsis were identified during the interim results meeting as the key nosocomial infections of interest.

Summary Statistics

The number and percentage of subjects who have at least one suspected nosocomial infection during the 10 day treatment period will be summarised by treatment group. In addition the total number of infections (1, 2, 3, >3) and the type of infection will be summarised as frequency counts and percentages.

Following the interim analysis, nosocomial infections will also be summarised by age category.

Logistic Regression

The proportion of subjects who have at least one suspected nosocomial infection in each treatment group will be compared using a logistic regression. Covariates/ factors will be included in the model as described in Section 7.1**Error! Reference source not found.**. The odds ratio with associated 95% CI will be presented.

This analysis may not be performed if there are a low number of suspected nosocomial infections.

9.4. Transepidermal water loss (TEWL)

TEWL (as measured by a tewameter) readings were taken on Day 0, 2, 4, 6, 8 and 10. At each time point two readings were taken. The average of the two readings will be determined and used for the analysis described below.

TEWL and change in TEWL will be summarised by day, presenting the number of subjects, mean (with standard deviation) and median, minimum and maximum for each treatment group. Change from baseline (mean and 95% CI) in TEWL will also be plotted over time. Day will be presented on the x-axis and the change from baseline will be presented on the y-axis.

Change in TEWL will also be analysed using a MMRM following the methods specified in Section 9.1.

Following the interim analysis, investigation will also be conducted into the effect of age category on TEWL. A similar approach to that of the weight endpoint will be considered for the statistical analysis except all subjects will be included in the analysis rather than only subjects without oedema.

9.5. Skin redness

Skin redness (as assessed by the colorimeter) readings for L, a and b were taken on Day 0, 2, 4, 6, 8 and 10. At each timepoint two readings of L, a and b were taken. The average

of the two readings will be determined and used for the analysis described below. L, a and b will be summarised separately.

Skin redness and change in skin redness will be summarised by day, presenting the number of subjects, mean (with standard deviation) and median, minimum and maximum for each treatment group. Change from baseline (mean and 95% CI) in skin redness will also be plotted over time. Day will be presented on the x-axis and the change from baseline will be presented on the y-axis.

Change in skin redness will also be analysed using a MMRM following the methods specified in Section 9.1.

Following the interim analysis, investigation will also be conducted into the effect of age category on skin redness. A similar approach to that of the weight endpoint will be considered for the statistical analysis except all subjects will be included in the analysis rather than only subjects without oedema.

9.6. Skin Appearance Score

Derivations

Skin appearance score is collected on Days 0, 2, 4, 6, 8 and 10. The total score will be summarised as described below, the individual components which make up the total score will not be summarised.

Summary Statistics

The absolute and change from baseline values for skin appearance score will be summarised by day, presenting the number of subjects, mean (with standard deviation) and median, minimum and maximum for each treatment group. The summaries will also present summary statistics of the endpoint on the last day the subject was in the study.

Change from baseline (mean and 95% CI) for skin appearance score will also be plotted over time. Day will be presented on the x-axis and the change from baseline will be presented on the y-axis. The mean at each day will be presented with an associated 95% CI.

Mixed model repeated measures analysis

Change from baseline in skin appearance score will be analysed using a MMRM following the methods specified in Section 9.1.

Following the interim analysis, investigation will also be conducted into the effect of age category on skin appearance score. A similar approach to that of the weight endpoint will be considered for the statistical analysis except all subjects will be included in the analysis rather than only subjects without oedema.

Further analysis

Non-linear modelling and latent growth curves may be considered if deemed appropriate.

9.7. SCORDoK Score

Skin condition as measured by the modified SCORDoK score is collected on Days 0, 2, 4, 6, 8 and 10.

SCORDoK scores will be provided by icddr,b, GSK and the Denmark SCORDoK team but only scores from iccdr,b will be included in the outputs specified in this RAP.

A summary table showing the number and percent of subjects with each type of skin manifestation will be produced by day and treatment group. The table will also show the grade for ichtyosiform skin changes, lichenoid skin changes and bullae-erosions-desquamations.

Further analysis of the SCORDoK score may be carried out by the Denmark SCORDoK team but this work is out of score for this RAP.

Following the interim analysis, SCORDoK will also be summarised by age category.

9.8. Plasma Fatty Acids

Fatty acid (FA) data will not be included in the interim analysis as the results will not be available until the end of the study. The fatty acid data will be analysed as part of the final analysis.

Fatty acids at Day 0 and Day 10 will be summarised along with the change in FA and percentage change in FA at Day 10. The data displays and exploratory analysis of the FA data will be performed by Metabolon Inc, and further analyses may be performed by the

Statistics group of the GSK subsidiary Cellzome. GSK Statistics and Programming team will assist in these analyses as needed including providing any datasets or covariates for statistical analyses.

9.9. Plasma C-Reactive Protein (CRP)

CRP at Day 0 and Day 10 will be summarised along with the change in CRP and percentage change in CRP at Day 10. The output will present the number of subjects, mean (with standard deviation) and median, minimum and maximum for each treatment group.

The number and percentage of subjects with positive and negative CRP levels will also be summarised, where positive is a CRP value > 0.5 mg/dL and negative is a CRP value ≤ 0.5mg/dL.

9.10. Pro-inflammatory cytokines

Pro-inflammatory cytokines (including IL-1ß, IL-2, IL-4, IL-5, IL-6, IL-7, IL-8, IL-10, IL-12p70 , IL-13, IL-17, TNF-ἀ, G-CSF, GM-CSF, IFN-γ, MCP-1 (MCAF) and MIP-1β) will be summarised at Day 0 and 10 along with the change in pro-inflammatory cytokines and percentage change in pro-inflammatory cytokines at Day 10. The output will present the number of subjects, mean (with standard deviation) and median, minimum and maximum for each treatment group.

For laboratory values that are above or below the lower limit of quantification or out of range (OOR), that have character values starting with “<” or “>,” numeric values will be derived using the following rules, where, for example, IL5I is the character result in standard units, and IL5 is the numeric version of the result:

if IL5I > x then IL5 = x + 1, where x is an integer >= 1.
if IL5I > x.y then IL5 = x.y +0.1, where x, y are integers.
if IL5I > x.yz then IL5 = x.yz + 0.01, where x, y, z are integers
etc.

if IL5I “< x” then IL5 = x - 1, where x is an integer >= 1.
if IL5I “< x.y” then IL5 = x.y - 0.1, where x, y are integers.
if IL5I “< x.yz” then IL5 = x.yz - 0.01, where x, y, z are integers
etc.

10. SAFETY ANALYSES

All safety tables, listings and figures will use the Safety population unless specified otherwise.

10.1. Adverse Events

A listing of all new problems during the hospital stay, including nosocomial infections, will be produced.

In addition, any ongoing illnesses, as collected on the daily monitoring chart, will be listed.

10.2. Concomitant Medications

A listing of all antibiotics given during the study will be produced. Antibiotics that were taken prior to the start of the study will not be included.

The listing will also include the duration of the concomitant medication prescription. This will be reported as the total number of days prescribed. Following the interim analysis discussions, a separate listing will be provided restricting the duration to the number of days that the concomitant medication taken during the study. For example, if a child on day 8 was prescribed 14-days' worth of concomitant medication then the first listing would record this as '14' and the second would record this as '3' i.e. the concomitant medication was taken on study day 8, 9 and 10.

10.3. Laboratory Measurements

A listing of the complete blood count and other biochemical / serological investigations laboratory data collected during the study will be produced.

10.4. Vitals Signs

Pulse, temperature, respiratory rate and mental status (Normal / irritable / lethargic) were collected three times a day. This data will be listed.

Supplemental Table 1.
Emollient (sunflower seed oil) composition

Product description:
Refined sunflower oil is a vegetable oil of non-GM origin and is non-hydrogenated.

Compliance with Food legislation:
This product complies with all current and relevant requirements, which are set by EU food legislation. Cargill's "Standards on Unwanted Components" is available upon request and contains legal limits for contaminants, quality and food safety parameters.

TECHNICAL SPECIFICATIONS [1)]				
		**Min**	**Max**	***Reference method*** [2)]
**Sensory**:				
Taste	-	bland		*Cargill Internal method*
Appearance at room temperature	-	clear		*Cargill Internal method*
**Chemical**:				
Free Fatty Acid, as oleic	%	-	0.10	*EN-ISO 660:2009*
Peroxide Value, at bottling	meq/kg	-	2.0	*ISO 3960:2007*
Moisture Content	%	-	0.10	*ISO 8534:2007*
Colour Lovibond 5.25”	Red	-	2.0	*ISO 15305:1998*
**Fatty Acid Composition:**		*EN-ISO 5509:2000 & EN-ISO 5508:1995*		
C16:0	%	5.0	7.6	
C18:0	%	2.7	6.5	
C18:1 (total)	%	14.0	39.4	
C18:2 (total)	%	48.3	74.0	
C18:3 (total)	%	-	0.5	
Trans fatty acids (total)	%	-	2.0	

1) Analyses are done by our refineries/ suppliers before reception of the oils in the bottling plant
2) Cargill reserves the right to use internal analytical method that is in compliance with the International Reference Method

Supplemental Table 2: Skin condition score assessment

Score	Skin Description	Day 0		Day 2		Day 4		Day 6		Day 8		Day 10	
		Area (%)	Locations	Area (%)	Locations	Area (%)	Locations	Area (%)	Locations	Area (%)	Locations	Area (%)	Locations
0	Normal, no sign of dry skin or scaling												
1	Skin dry, no visible scales												
2	Skin dry with fine scales												
3	Skin dry with scales and fissures, no erythema												
4	Dry skin with scales, fissures and erythema												
5	Dry skin with scales, fissures, erythema and focal areas of crusting and/or oozing												
6	Dry skin with scales, fissures, 10% body surface area with erythema, crusting and/or oozing												
7	Nearly entire skin is erythematous and/or oozing												
8	Vesicles or pustules present												
	**Total Score**												

Score of all calculation method: For skin scoring we multiplied the area (%) of involvement with the corresponding category's score. The each % of area correspond the score of that category. For example: in line number three, ' Skin dry with fine scales' corresponds the socre '2', so each % of skin area with such characteristic will get 'X' % x 2 score. Total skin appearance score will be the summation available score in individual column.

Location code and % of area:

Location	Code	Area (%)	Location	Code	Area (%)
Head, anterior	HA	9	Upper trunk, anterior	UTA	9
Head, Posterior	HP	9	Upper trunk, Posterior	UTP	6.5
Rt . Upper arm, Anterior	Rt.UAA	2.25	Lower trunk, anterior	LTA	9
Rt. Upper arm, Posterior	Rt.UAP	2.25	Lower trunk, posterior	LTP	6.5
Rt. Lower arm, Anterior	Rt.LAA	2.25	Lt. Buttock	Lt.BT	2.5
Rt. Lower arm,Posterior	Rt. LAP	2.25	Rt. Buttock	Rt.BT	2.5
Lt . Upper arm, Anterior	Lt.UAA	2.25	Rt. lower leg, anterior	Rt.LLA	3.5
Lt. Upper arm, posterior	Lt.UAP	2.25	Rt. lower leg, posterior	Rt.LLP	3.5
Lt. Lower arm, Anterior	Lt. LAA	2.25	Lt. upper leg, anterior	Lt.ULA	3.5
Lt. Lower arm,Posterior	Lt. LAP	2.25	Lt. upper leg, posterior	Lt.ULP	3.5
Rt. Upper leg, anterior	Rt.ULA	3.5	Lt. lower leg, anterior	Lt.LLA	3.5
Rt. Upper leg, posterior	Rt.ULP	3.5	Lt. lower leg, posterior	Lt.LLP	3.5
**Total = 100 %**					
